# Impact of cardiovascular disease and cardiovascular risk factors in hospitalised COVID-19 patients

**DOI:** 10.1007/s12471-021-01572-9

**Published:** 2021-04-16

**Authors:** L. S. D. Jewbali, J. Hoogervorst-Schilp, E. Belfroid, C. W. Jansen, F. W. Asselbergs, H. J. Siebelink

**Affiliations:** 1 Department of Cardiology and Intensive Care Medicine, Erasmus University Medical Center, Rotterdam, The Netherlands; 2 Knowledge Institute of Medical Specialists, Utrecht, The Netherlands; 3 Netherlands Society of Cardiology, Utrecht, The Netherlands; 4 Department of Cardiology, University Medical Center Utrecht, Utrecht, The Netherlands; 5 Department of Cardiology, Leiden University Medical Center, Leiden, The Netherlands

**Keywords:** COVID-19, Cardiovascular risk, Cardiovascular disease, Outcome

## Abstract

**Background:**

Hospitalised COVID-19 patients with underlying cardiovascular disease (CVD) and cardiovascular risk factors appear to be at risk of poor outcome. It is unknown if these patients should be considered a vulnerable group in healthcare delivery and healthcare recommendations during the COVID-19 pandemic.

**Methods:**

A systematic literature search was performed to answer the following question: In which hospitalised patients with proven COVID-19 and with underlying CVD and cardiovascular risk factors should doctors be alert to a poor outcome? Relevant outcome measures were mortality and intensive care unit admission. Medline and Embase databases were searched using relevant search terms until 9 June 2020. After systematic analysis, 8 studies were included.

**Results:**

Based on the literature search, there was insufficient evidence that CVD and cardiovascular risk factors are significant predictors of mortality and poor outcome in hospitalised patients with COVID-19. Due to differences in methodology, the level of evidence of all studies was graded ‘very low’ according to the Grading Recommendations Assessment, Development and Evaluation methodology. It is expected that in the near future, two multinational and multicentre European registries (CAPACITY-COVID and LEOSS) will offer more insight into outcome in COVID-19 patients.

**Conclusion:**

This literature review demonstrated there was insufficient evidence to identify CVD and cardiovascular risk factors as important predictors of poor outcome in hospitalised COVID-19 patients. However, patients with CVD and cardiovascular risk factors remain vulnerable to infectious disease outbreaks. As such, governmental and public health COVID-19 recommendations for vulnerable groups apply to these patients.

**Supplementary Information:**

The online version of this article (10.1007/s12471-021-01572-9) contains supplementary material, which is available to authorized users.

## Clinical question

*In which hospitalised patients with proven COVID-19 and with underlying cardiovascular disease and cardiovascular risk factors should doctors be alert to a poor outcome?*

## Introduction

Hospitalised COVID-19 patients with pre-existing cardiovascular disease (CVD) and risk factors for CVD seem to have a worse outcome. CVD risk factors appear to affect the immune function and thus relate indirectly to the prognosis in COVID-19 patients [1, 2]. Identifying COVID-19 patients at risk of worse outcome can tailor healthcare delivery and healthcare measures such as vaccination policy. It can also increase patient awareness regarding risk factors for COVID-19.

## Methods

A review of the literature was performed to answer the following question: Which independent prognostic factors (cardiovascular risk factors or CVD) strongly predict a poor outcome of COVID-19, independent of other factors? This question was structured in PICO format.

**P**opulation: All patients with proven COVID-19
**I**ntervention: Presence of one of the following prognostic factors: cardiovascular risk factors such as smoking, obesity, hypercholesterolaemia, hypertension, diabetes mellitus (insulin resistance, non-alcoholic steatohepatitis), CVD, cardiovascular history (arrhythmias, coronary artery disease, heart failure, valvular heart disease)
**C**omparison: Absence of the prognostic factors
**O**utcome: Mortality (crucial), intensive care unit (ICU) admission (crucial), hospital admission, length of stay, thromboembolic complications (pulmonary embolism, stroke, transient ischaemic attack)
Timing: Admission to hospital, admission to ICU, during hospital stay, at home
Setting: In-hospital, pre-hospital
Confounder: Age

### Relevant outcome measures

Mortality and ICU admission were considered critical outcome measures for decision-making, whereas the other outcome measures were considered important outcome measures. Definitions of outcome measures from the studies were used. Minimal clinically relevant differences for the outcome measures were not defined.

### Search and select

The databases Medline (via Ovid) and Embase (via www.embase.com) were searched using relevant search terms until 9 June 2020. The systematic literature search resulted in 567 hits (see Table S1 in the Electronic Supplementary Material for search strategy). We did not find any studies investigating the impact of a multivariable cardiovascular prognostic model. Studies developing and/or validating a multivariable prognostic model were selected based on the following criteria: systematic review, randomised controlled trial or observational study (cohort study) assessing the longitudinal relation between cardiovascular risk factors (smoking, obesity, hypercholesterolaemia, hypertension, diabetes mellitus), CVD and cardiovascular history (measured at hospital admission/during hospital stay) with mortality, ICU admission, hospital admission, length of stay, thromboembolic complications (measured at endpoint) in patients with proven COVID-19. Age was considered a confounder that had to be included in the multivariable models.

Initially, 45 studies were selected based on title and abstract screening. After reading the full text, 37 studies were excluded (excluded studies are listed in Table S2 in the Electronic Supplementary Material), resulting in the inclusion of 8 studies in the literature review. A brief overview of study characteristics of the included studies is shown in Tab. 1. The assessment of the risk of bias is summarised in the risk of bias table (see Table S3 in the Electronic Supplementary Material). In many studies, populations, measurement of factors and selection methods of factors were not well described.

**Table 1:** Study characteristics of included studies

Study (first author name, reference)	Population	*N*	Age in years; median (IQR)	Inclusion period	Follow-up	Method	Outcome
Chen [3]	Hospitalised COVID-19 patients, 575 hospitals, China	1590	Not reported	Admission to hospital until 31 Jan 2020 (time of admission to hospital not reported)	Not reported	Multivariate Cox regression model; included prognostic factors selected based on univariable analyses; nomogram developed based on backward step-down selection	Mortality
Cummings [6]	Hospitalised COVID-19 patients (≥18 years), critically ill with acute hypoxaemic respiratory failure, 2 hospitals, USA	257	62 (51–72)	Admission to hospital between 2 Mar–1 Apr 2020; candidate factors measured during hospital stay (collected from medical records)	28 Apr 2020	Multivariate Cox regression model; included prognostic factors considered relevant to in-hospital mortality by the authors	In-hospital mortality
Gao [7]	Hospitalised COVID-19 patients, 1 hospital, China	2877	Not reported for total group	Admission to hospital between 5 Feb–15 Mar 2020; candidate factors measured during hospital stay (collected from medical records)	1 Apr 2020	Multivariable Cox proportional hazards model; reason of selection of included prognostic factors in multivariable model not described	In-hospital mortality
Giacomelli [4]	Hospitalised COVID-19 patients (≥18 years), 1 hospital, Italy	233	61 (50–72)	Admission to hospital between 21 Feb–19 Mar 2020; candidate factors measured during hospital stay (collected from medical records)	20 Apr 2020	Multivariable Cox proportional hazards model; included prognostic factors selected based on univariable analyses	Mortality
Klang [8]	Hospitalised COVID-19 patients (≥18 years), 5 hospitals, USA	3406	Not reported for total group	Admission to hospital between 1 Mar–17 May 2020; candidate factors measured during hospital stay (collected from medical records)	Not reported	Multivariable logistic regression model, adjusted for age decile, male sex, CAD, CHF, hypertension, DM, hyperlipidaemia, CKD, history of cancer, smoking (past or present), BMI 30–40 kg/m^2^, BMI ≥40 kg/m^2^ and race; included prognostic factors selected based on univariable analyses; no validation reported	In-hospital mortality
Palaiodimos [9]	Hospitalised COVID-19 patients, 1 hospital, USA	200	64 (50–73.5)	Admission to hospital between 9–22 Mar 2020; candidate factors measured during hospital stay (collected from medical records)	3‑weeks follow-up: 12 Apr 2020	Multivariate logistic regression model; 3 models used (model 1: BMI and age; model 2: all variables with significant univariate associations; model 3: variables of model 2 plus clinically significant variables that did not show a significant univariate association); no validation reported	In-hospital mortality
Petrilli [10]	Admitted to hospital and non-admitted COVID-19 patients, >260 outpatient office sites and 4 acute care hospitals, USA	5279 (2441 admitted to hospital)	Tested population: 54 (38–66); admitted population: 63 (51–74)	Patients tested between 1 Mar–8 Apr 2020; candidate factors measured during hospital stay (collected from medical records)	5 May 2020	Multivariable logistic regression model; predictors selected based on published literature and authors’ clinical experience with COVID-19 patients	Inpatient hospital admission, discharge to hospice or death among those admitted to hospital
Wang [5]	Hospitalised COVID-19 patients (>60 years), 1 hospital, China	339	69 (65–76)	Admission to hospital between 1 Jan–6 Feb 2020; candidate factors measured during hospital stay (collected from medical records)	4 weeks from last admission	Multivariate Cox regression model; included prognostic factors selected based on univariable analyses; no validation reported	Mortality

*IQR* interquartile range, *CAD* coronary artery disease, *CHF* congestive heart failure, *DM* diabetes mellitus, *CKD* chronic kidney disease, *BMI* body mass index

The level of evidence was assessed according to the Grading Recommendations Assessment, Development and Evaluation (GRADE) methodology (www.gradeworkinggroup.org).

### Description of studies

**Chen** [3], **Giacomelli** [4] and **Wang** [5] assessed candidate factors during hospital stay and mortality as endpoint. **Cummings** [6], **Gao** [7], **Klang** [8] and **Palaiodimos** [9] assessed candidate factors during hospital stay and in-hospital mortality as endpoint. **Petrilli** [10] assessed candidate factors during hospital stay and discharge to hospice or death among those patients admitted to hospital as endpoint. Multivariable models showing associations between predefined candidate prognostic factors and outcome were reported. Only **Chen** [3] internally validated the risk factors by establishing a nomogram based on the results of the multivariate analysis. It was decided to include non-validated studies in the literature review for this outcome as well, as risk of bias was moderate in the study by Chen.

## Results

All studies described models predicting mortality. Only **Petrilli** [10] also reported on a model predicting hospital admission. Tab. 2 shows the 8 model designs. The selected studies did not report on the prespecified outcome measures ICU admission, in-hospital length of stay and thromboembolic complications. Therefore, apart from mortality, other outcomes cannot be described.

**Table 2:** Reported prognostic models for mortality

Study (first author name, reference)	Mortality (*n*/*N* (%))	Outcome	Included prognostic factors
Chen [3]	50/1590 (3.1%)	Mortality	Age, coronary heart disease, cerebrovascular disease, dyspnoea, procalcitonin, aspartate aminotransferase, total bilirubin, creatinine
Cummings [6]	101/257 (39.0%)	Time from hospital admission to in-hospital mortality	Age, sex, symptom duration before hospital presentation, hypertension, chronic cardiac disease, COPD, DM, IL‑6 concentrations, D‑dimer concentrations
Gao [7]	56/2877 (1.9%)	All-cause mortality during hospitalization	Hypertension, age, sex, DM, myocardial infarction, treatment with PCI or CABG, renal failure, chronic heart failure, asthma, COPD, stroke
Giacomelli [4]	48/233 (20.6%)	Mortality (censoring date: 20 Apr 2020)	Age, sex, obesity, being treated with ≥1 antihypertensive agent, disease severity, presence of anaemia, lymphocyte count, D‑dimer, C‑reactive protein, creatinine, creatine kinase
Klang [8]	1136/3406 (33.4%)	In-hospital mortality	Age, sex, comorbidities (CAD, CHF, hypertension, DM, hyperlipidaemia, CKD, cancer), obesity, smoking status
Palaiodimos [9]	48/200 (24%)	In-hospital mortality	Age, BMI, heart failure, CAD, DM, CKD or end-stage renal disease, COPD, current or former smoker
Petrilli [10]	665/2741 (24.3%)	(1) Admission to hospital (2) Mortality (only admitted patients in analysis)	Age, BMI, sex, ethnicity, smoking status, CAD, heart failure, hypertension, DM, asthma or COPD, CKD, cancer
Wang [5]	65/339 (19.2%)	Mortality	Age, CVD, cerebrovascular disease, COPD

*COPD* chronic obstructive pulmonary disease, *DM* diabetes mellitus, *IL* interleukin, *PCI* percutaneous coronary intervention, *CABG* coronary artery bypass grafting, *CAD* coronary artery disease, *CHF* congestive heart failure, *CKD* chronic kidney disease, *BMI* body mass index, *CVD* cardiovascular disease

### Mortality predicted by prognostic factors measured during hospital stay

As the reported models included different outcome measures and different prognostic factors, results could not be pooled. Because the models were not validated, only results regarding factors included in at least two studies are discussed. Tab. 3 shows the relevance of the reported associations for mortality.

**Table 3:** Relevance of prognostic factors for mortality

	Statistically significant for mortality in respective study (Yes/No)								
	Chen [3]	Cummings [6]	Gao [7]	Giacomelli [4]	Klang [8] (age ≤50 years)	Klang [8] (age >50 years)	Palaiodimos [9]	Petrilli [10]	Wang [5]
Predictor	HR (95% CI)	HR (95% CI)	HR (95% CI)	HR (95% CI)	OR (95% CI)	OR (95% CI)	OR (95% CI)	HR (95% CI)	HR (95% CI)
Age, years	**Yes** <65 y: ref 65–74 y: 3.43 (1.24–9.5) ≥75 y: 7.86 (2.44–25.35)	**Yes** Per 10 y: 1.39 (1.29–1.57)	**Yes** Per year: 1.06 (1.04–1.09)	**Yes** Per 10 y: 2.08 (1.48–2.92)	**Yes** Per 10 y: 3.0 (1.9–4.8)	**Yes** Decile: 1.7 (1.6–1.8)	**Yes** Quartiles: 1.73 (1.13–2.63)	**Yes** 19–44 y: ref 45–54 y: 1.12 (0.80–1.60) 55–64 y: 2.04 (1.50–2.80) 65–74 y: 2.88 (2.46–4.80) ≥75 y: 3.46 (2.46–4.80)	**Yes** 1.86 (1.06–3.26)
BMI, kg/m^2^	–	No <40: ref ≥40: 0.76 (0.4–1.47)	–	**Yes** <30: ref ≥30: 3.04 (1.42–6.49)	**Yes** <30: ref 30–40: 1.1 (0.5–2.3) ≥40: 5.1 (2.3–11.1)	**Yes** >30: ref 30–40: 1.1 (0.9–1.3) ≥40: 1.6 (1.2–2.3)	**Yes** 25–34: ref <25: 1.37 (0.52–3.64) ≥35: 3.78 (1.45–9.83)	**Yes** <25: ref 25–30: 0.91 (0.74–1.11) 30–40: 1.02 (0.82–1.27) ≥40: 1.41 (0.98–3.02) Unknown: 1.85 (1.13–3.02)	–
Smoking	–	–	–	–	No 1.7 (0.8–3.8)	No 1.0 (0.8–1.2)	No 0.83 (0.37–1.87)	**Yes** Never: ref Former: 1.13 (0.93–1.37) Current: 0.90 (0.61–1.31) Unknown: 1.56 (1.26–1.93)	–
Hypertension (yes/no)	–	No 1.58 (0.89–2.81)	**Yes** 2.00 (1.13–3.54)	–	No 0.5 (0.2–1.1)	No 1.1 (0.9–1.3)		No 0.94 (0.76–1.16)	–
Diabetes mellitus (yes/no)	–	No 1.31 (0.81–2.10)	–	–	No 1.3 (0.7–2.6)	**Yes** 1.4 (1.2–1.7)	No 1.16 (0.55–2.44)	No 1.10 (0.93–1.31)	–
Coronary artery disease or congestive heart failure (yes/no)	–	**Yes** 1.76 (1.08–2.86)	–	–	–	–	–	–	–
Coronary artery disease (yes/no)	**Yes** 4.28 (1.14–16.13)	–	–	–	No 0.6 (0.2–2.1)	**Yes** 1.3 (1.1–1.6)	No 1.53 (0.54–4.34)	No 1.12 (0.92–1.36)	–
Cerebrovascular disease (yes/no)	**Yes** 3.1 (1.07–8.94)	–	–	–	–	–	–	–	No 1.38 (0.65–2.93)
Heart failure (yes/no)	–	–	**Yes** 3.3 (1.33–8.19)	–	**Yes** 4.0 (1.6–10.4)	No 1.0 (0.8–1.3)	No 1.43 (0.50–4.06)	**Yes** 1.77 (1.43–2.20)	–

*HR* hazard ratio, *CI* confidence interval, *OR* odds ratio, *BMI* body mass index

#### Body mass index

Starting with a high level of evidence for prognostic studies, the level of evidence regarding the outcome measure mortality was downgraded by one level because of risk of bias (some patients still under treatment at end of study), by one level because of indirectness (studies not validated) and by one level for imprecision (wide confidence intervals) to ‘very low’.

#### Smoking

Starting with a high level of evidence for prognostic studies, the level of evidence regarding the outcome measure mortality was downgraded by one level because of risk of bias (some patients still under treatment at end of study), by one level because of indirectness (studies not validated) and by one level for imprecision (wide confidence intervals) to ‘very low’.

#### Hypertension

Starting with a high level of evidence for prognostic studies, the level of evidence regarding the outcome measure mortality was downgraded by one level because of risk of bias (some patients still under treatment at end of study), by one level because of indirectness (studies not validated) and by one level for imprecision (wide confidence intervals, too many prognostic factors included in relation to number of events) to ‘very low’.

#### Diabetes mellitus

Starting with a high level of evidence for prognostic studies, the level of evidence regarding the outcome measure mortality was downgraded by one level because of risk of bias (some patients still under treatment at end of study), by one level because of indirectness (studies not validated) and by one level for imprecision (wide confidence intervals) to ‘very low’.

#### Coronary artery disease or congestive heart failure

Starting with a high level of evidence for prognostic studies, the level of evidence regarding the outcome measure mortality was downgraded by one level because of risk of bias (some patients still under treatment at end of study), by one level because of indirectness (studies not validated) and by one level because of inconsistency of results to ‘very low’.

#### Cerebrovascular disease

Starting with a high level of evidence for prognostic studies, the level of evidence regarding the outcome measure mortality was downgraded by one level because of risk of bias (some patients still under treatment at end of study), by one level because of indirectness (studies not validated), by one level because of inconsistency of results and by one level because of imprecision (low number of included patients) to ‘very low’.

#### Heart failure

Starting with a high level of evidence for prognostic studies, the level of evidence regarding the outcome measure mortality was downgraded by one level because of risk of bias (some patients still under treatment at end of study), by one level because of indirectness (studies not validated), by one level because of inconsistency of results and by one level because of imprecision (low number of included patients) to ‘very low’.

## Conclusion

### Mortality predicted by prognostic factors measured during hospital stay

#### Body mass index

It is unsure whether body mass index (BMI) is a predictor of mortality in COVID-19 patients admitted to hospital, adjusted for age*. Sources: Cummings, Giacomelli, Klang, Palaiodimos, Petrilli ***(very low GRADE level)**.

#### Smoking

It is unsure whether smoking is a predictor of mortality in COVID-19 patients admitted to hospital, adjusted for age*. Sources: Klang, Palaiodimos, Petrilli ***(very low GRADE level)**.

#### Hypertension

It is unsure whether hypertension is a predictor of mortality in COVID-19 patients admitted to hospital, adjusted for age*. Sources: Cummings, Gao, Klang, Petrilli ***(very low GRADE level)**.

#### Diabetes mellitus

It is unsure whether diabetes is a predictor of mortality in COVID-19 patients admitted to hospital, adjusted for age*. Sources: Cummings, Klang, Palaiodimos, Petrilli ***(very low GRADE level)**.

#### Coronary artery disease

It is unsure whether coronary artery disease or congestive heart failure is a predictor of mortality in COVID-19 patients admitted to hospital, adjusted for age*. Sources: Chen, Cummings, Klang, Palaiodimos, Petrilli ***(very low GRADE level)**.

#### Cerebrovascular disease

It is unsure whether cerebrovascular disease is a predictor of mortality in COVID-19 patients admitted to hospital, adjusted for age*. Sources: Chen, Wang ***(very low GRADE level)**.

#### Heart failure

It is unsure whether heart failure is a predictor of mortality in COVID-19 patients admitted to hospital, adjusted for age*. Sources: Klang, Palaiodimos, Petrilli ***(very low GRADE level)**.

#### Other cardiovascular risk factors, cardiovascular disease or cardiovascular history

We cannot conclude which other cardiovascular risk factors, CVD or cardiovascular history can predict hospital admission or mortality in COVID-19 patients due to a lack of studies on multivariable models taking age into account** (no GRADE level)**.

In conclusion, based on this literature review, there was insufficient evidence that underlying CVD and cardiovascular risk factors are predictors of mortality in hospitalised COVID-19 patients. Populations, measurement of factors and selection methods of factors were not well described in many studies. Even though BMI could statistically be considered a risk factor for mortality, this was not supported by the level of evidence. Risk of bias, indirectness and imprecision according to the GRADE methodology led to downgrading the levels of evidence for the prognostic factors to ‘very low’.

## Discussion

The present literature review, up until 9 June 2020, demonstrated that there was insufficient evidence that CVD and cardiovascular risk factors have a significant effect on poor outcome in hospitalised COVID-19 patients. Studies validating prediction models for CVD are needed; once these become available, they can be added to this review of the literature.

### CAPACITY registry

The same research question was also studied by the Cardiac complicAtions in Patients With SARS Corona vIrus 2 regisTrY (CAPACITY registry), an international initiative to evaluate the role of CVD in hospitalised patients with COVID-19 [11]. In August 2020, 61 hospitals from 13 countries contributed to the data collection. The data from 40% of the hospitalised Dutch patients were entered into the database and the results were published in March 2021 [12].

Data from 5500 Dutch patients were analysed to answer the research question. Basic characteristics demonstrated that patients with CVD were older and had more cardiovascular risk factors and comorbidities. They developed acute kidney injury during hospitalisation more frequently and were more often diagnosed with chronic kidney disease. Older age, male gender and frailty seemed to have predictive value.

Combining data from the Dutch CAPACITY registry [12] with those from the Lean European Open Survey on SARS-CoV-2-Infected patients (LEOSS) registry showed a significant association between New York Heart Association class III/IV heart failure and in-hospital mortality for patients with cardiovascular comorbidity, after adjustment for age, gender, myocardial infarction, hypertension, chronic kidney disease, chronic obstructive pulmonary disease and diabetes mellitus.

### Recommendations

In general, patients with CVD and cardiovascular risk factors are considered to be vulnerable to infectious disease outbreaks. Based on our literature review and the results from the CAPACITY registry, there is no reason not to deem them vulnerable to COVID-19. The data indicate that these patients are older and have more comorbidities and suggest that older age, male gender and frailty are predictors of mortality. Of the patients who died of COVID-19, most had a history of CVD (See Box 1).

#### Box 1 Recommandations

- Regard patients with CVD and cardiovascular risk factors as vulnerable to COVID-19 and at risk of poor outcome.
- Be aware that patients with heart failure may have a greater risk of poor outcome and mortality.
- Healthcare professionals delivering care to patients with CVD should follow COVID-19 guidelines for vulnerable groups.
- Patients with CVD and cardiovascular risk factors should comply with the governmental and public health COVID-19 recommendations and advice for vulnerable groups. Implementation is expected to be high and should bear no extra costs since it is embedded in the current governmental policies.

### Gaps in evidence

The initial research question remains unanswered: In which hospitalised patients with proven COVID-19 and with underlying CVD and cardiovascular risk factors should doctors be alert to a poor outcome?

## Supplementary Information

Table S1 Literature search strategy

Table S2 Excluded studies

Table S3 Quality assessment and risk of bias
