# Supplementary material for: Impact of cardiovascular disease and cardiovascular risk factors in hospitalised COVID-19 patients
Source: Neth Heart J. 2021 Apr 16;29(Suppl 1):13–9. doi: 10.1007/s12471-021-01572-9 (PMC8050809; doi:10.1007/s12471-021-01572-9)
Supplement: Supplementary file 1 — Table S1 Literature search strategy [file 12471_2021_1572_MOESM1_ESM.docx]

**Table S1 Literature search strategy**

**Ovid/Medline**

1 ((exp Coronavirus/ or Coronavirus Infections/ or pneumonia virus*.ti,ab,kf. or cov.ti,ab,kf.) and ((outbreak or wuhan).ti,ab,kf. or novel.af. or '19'.ti,ab,kf. or '2019'.ti,ab,kf. or epidem*.af. or epidemy.af. or epidemic*.af. or pandem*.af. or new.ti,ab,kf.)) or (coronavirus* or 'corona virus*' or ncov or '2019ncov' or 'covid19' or "covid 19" or "sars cov 2" or 'sars2' or "ncov 2019" or "sars coronavirus 2" or "sars corona virus 2" or "severe acute respiratory syndrome cov 2" or "severe acute respiratory syndrome cov2" or "severe acute respiratory syndrome cov*").ti,ab,kf. (34799)

2 limit 1 to dt="20191201-20220101" (22129)

3 exp Cardiovascular Diseases/ or angiocardiopathy.ti,ab,kf. or angiocardiovascular disease.ti,ab,kf. or hypovolemia.ti,ab,kf. or ((cardiovascular or heart or cardiac or coronary) adj3 (complication* or disorder* or disturbance* or lesion* or syndrome* or event* or risk* or history or failure or overload or dysfunction or arrest or aneurysm* or anomal* or atheroscleros* or calcification* or constriction* or dissection or obstruction or occlusion or perforation or thrombos*)).ti,ab,kf. or exp Smoking/ or smoking.ti,ab,kf. or exp Obesity/ or adipose tissue hyperplasia.ti,ab,kf. or adipositas.ti,ab,kf. or adiposity.ti,ab,kf. or corpulency.ti,ab,kf. or fat overload syndrome.ti,ab,kf. or obesitas.ti,ab,kf. or obesity.ti,ab,kf. or overweight.ti,ab,kf. or obese.ti,ab,kf. or exp hypercholesterolemia/ or cholesteremia.ti,ab,kf. or cholesterinemia.ti,ab,kf. or cholesterolemia.ti,ab,kf. or hypercholesteremia.ti,ab,kf. or hypercholesterinaemia.ti,ab,kf. or hypercholesterinemia.ti,ab,kf. or hypercholesterolaemia.ti,ab,kf. or hypercholesterolemia.ti,ab,kf. or exp Hypertension/ or high blood pressure.ti,ab,kf. or hypertens*.ti,ab,kf. or exp Diabetes mellitus/ or diabetes.ti,ab,kf. or diabetic.ti,ab,kf. or insulin resistanc*.ti,ab,kf. or exp Non-alcoholic fatty liver disease/ or non alcoholic steato-hepatitis.ti,ab,kf. or non-alcoholic steatohepatitis.ti,ab,kf. or non-alcoholic steatosis hepatitis.ti,ab,kf. or non-alcoholic steatotic hepatitis.ti,ab,kf. or nonalcoholic fatty liver inflammation.ti,ab,kf. or nonalcoholic steato-hepatitis.ti,ab,kf. or nonalcoholic steatohepatitis.ti,ab,kf. or nonalcoholic steatosis hepatitis.ti,ab,kf. or nonalcoholic steatotic hepatitis.ti,ab,kf. or exp Arrhythmias, Cardiac/ or arrhythmia.ti,ab,kf. or ectopic heart rhythm.ti,ab,kf. or ectopic rhythm.ti,ab,kf. or heart aberrant conduction.ti,ab,kf. or arrhytmia.ti,ab,kf. or arrythmia.ti,ab,kf. or dysrhythmia.ti,ab,kf. or heart ectopic beat.ti,ab,kf. or heart ectopic ventricle contraction.ti,ab,kf. or heart rhythm disorder.ti,ab,kf. or atrioventricular junction arrhythmia.ti,ab,kf. or bradycardia.ti,ab,kf. or cardiac channelopath*.ti,ab,kf. or cardiopulmonary arrest*.ti,ab,kf. or commotio cordis.ti,ab,kf. or heart fibrillation.ti,ab,kf. or heart muscle conduction disturbance.ti,ab,kf. or heart palpitation.ti,ab,kf. or heart preexcitation.ti,ab,kf. or heart proarrhythmia.ti,ab,kf. or pacemaker failure*.ti,ab,kf. or parasystole.ti,ab,kf. or tachycardia.ti,ab,kf. or acute coronary syndrome.ti,ab,kf. or cardiac allograft vasculopathy.ti,ab,kf. or coronary bifurcation lesion.ti,ab,kf. or coronary subclavian steal syndrome.ti,ab,kf. or kounis syndrome.ti,ab,kf. or no reflow phenomenon.ti,ab,kf. (3642523)

4 2 and 3 (1382)

5 4 not ((exp animals/ or exp models, animal/) not humans/) not (letter/ or comment/ or editorial/) (1091)

6 (meta-analysis/ or meta-analysis as topic/ or (meta adj analy$).tw. or ((systematic* or literature) adj2 review$1).tw. or (systematic adj overview$1).tw. or exp "Review Literature as Topic"/ or cochrane.ab. or cochrane.jw. or embase.ab. or medline.ab. or (psychlit or psyclit).ab. or (cinahl or cinhal).ab. or cancerlit.ab. or ((selection criteria or data extraction).ab. and "review"/)) not (Comment/ or Editorial/ or Letter/ or (animals/ not humans/)) (449490)

7 (exp clinical trial/ or randomized controlled trial/ or exp clinical trials as topic/ or randomized controlled trials as topic/ or Random Allocation/ or Double-Blind Method/ or Single-Blind Method/ or (clinical trial, phase i or clinical trial, phase ii or clinical trial, phase iii or clinical trial, phase iv or controlled clinical trial or randomized controlled trial or multicenter study or clinical trial).pt. or random*.ti,ab. or (clinic* adj trial*).tw. or ((singl* or doubl* or treb* or tripl*) adj (blind$3 or mask$3)).tw. or Placebos/ or placebo*.tw.) not (animals/ not humans/) (1990319)

8 Epidemiologic studies/ or case control studies/ or exp cohort studies/ or Controlled Before-After Studies/ or Case control.tw. or (cohort adj (study or studies)).tw. or Cohort analy$.tw. or (Follow up adj (study or studies)).tw. or (observational adj (study or studies)).tw. or Longitudinal.tw. or Retrospective*.tw. or prospective*.tw. or consecutive*.tw. or Cross sectional.tw. or Cross-sectional studies/ or historically controlled study/ or interrupted time series analysis/ [Onder exp cohort studies vallen ook longitudinale, prospectieve en retrospectieve studies] (3449163)

9 5 and 6 (67)**Systematic reviews**

10 5 and 7 (78)

11 5 and 8 (205)

12 10 not 9 (60) **RCT’s**

13 11 not 10 not 9 (169) **Observationeel**

**Embase Session Results (9 Jun 2020)**

| No. | Query | Results |
| --- | --- | --- |
| #27 | #20 NOT #19 NOT #18 **Observationeel (P AND I)** | **282** |
| #26 | #19 NOT #18 **RCT’s (P AND I)** | **113** |
| #20 | #4 AND #12 | **381** |
| #19 | #4 AND #11 | **132** |
| #18 | #4 AND #5 **Systematic reviews (P AND I)** | **94** |
| #17 | #15 NOT #14 NOT #13 Observationeel (P AND I AND O) | **195** |
| #16 | #14 NOT #13 RCT (P AND I AND O) | **84** |
| #15 | #9 AND #12 | **268** |
| #14 | #9 AND #11 | **99** |
| #13 | #9 AND #10 SR (P AND I AND O) | **68** |
| #12 | 'major clinical study'/de OR 'clinical study'/de OR 'case control study'/de OR 'family study'/de OR 'longitudinal study'/de OR 'retrospective study'/de OR 'prospective study'/de OR 'comparative study'/de OR 'cohort analysis'/de OR ((cohort NEAR/1 (study OR studies)):ab,ti) OR (('case control' NEAR/1 (study OR studies)):ab,ti) OR (('follow up' NEAR/1 (study OR studies)):ab,ti) OR (observational NEAR/1 (study OR studies)) OR ((epidemiologic NEAR/1 (study OR studies)):ab,ti) OR (('cross sectional' NEAR/1 (study OR studies)):ab,ti) | **5969696** |
| #11 | ('clinical trial'/exp OR 'randomization'/exp OR 'single blind procedure'/exp OR 'double blind procedure'/exp OR 'crossover procedure'/exp OR 'placebo'/exp OR 'prospective study'/exp OR rct:ab,ti OR random*:ab,ti OR 'single blind':ab,ti OR 'randomised controlled trial':ab,ti OR 'randomized controlled trial'/exp OR placebo*:ab,ti) NOT 'conference abstract':it | **2399535** |
| #10 | ('meta analysis'/de OR cochrane:ab OR embase:ab OR psycinfo:ab OR cinahl:ab OR medline:ab OR ((systematic NEAR/1 (review OR overview)):ab,ti) OR ((meta NEAR/1 analy*):ab,ti) OR metaanalys*:ab,ti OR 'data extraction':ab OR cochrane:jt OR 'systematic review'/de) NOT (('animal experiment'/exp OR 'animal model'/exp OR 'nonhuman'/exp) NOT 'human'/exp) | **497183** |
| #9 | #8 NOT ('conference abstract'/it OR 'editorial'/it OR 'letter'/it OR 'note'/it) NOT (('animal experiment'/exp OR 'animal model'/exp OR 'nonhuman'/exp) NOT 'human'/exp) | **1027** |
| #8 | #3 AND #7 | **1310** |
| #7 | 'mortality'/exp OR 'mortality':ti,ab OR death*:ti,ab OR fatal*:ti,ab OR 'hospital admission'/exp OR 'hospital admission':ti,ab OR 'hospital admittance':ti,ab OR 'patient admission':ti,ab OR 'intensive care'/exp OR 'critical care':ti,ab OR 'intensive care':ti,ab OR 'intensive therapy':ti,ab OR 'length of stay'/exp OR 'length of stay':ti,ab OR 'infarction'/exp OR 'bloodless':ti,ab OR 'infarct*':ti,ab OR 'thromboembolic accident':ti,ab OR 'thromboembolism'/exp OR thromboembolism*:ti,ab OR 'lung embolism*':ti,ab OR 'pulmonary embolism*':ti,ab OR 'cerebrovascular accident'/exp OR cva:ti,ab OR 'cerebrovascular accident':ti,ab OR 'transient ischemic attack'/exp OR tia:ti,ab OR 'transient ischemic attack':ti,ab OR 'poor outcome':ti,ab | **4177433** |
| #6 | #4 AND #5 | **94** |
| #5 | ('meta analysis'/de OR cochrane:ab OR embase:ab OR psycinfo:ab OR cinahl:ab OR medline:ab OR ((systematic NEAR/1 (review OR overview)):ab,ti) OR ((meta NEAR/1 analy*):ab,ti) OR metaanalys*:ab,ti OR 'data extraction':ab OR cochrane:jt OR 'systematic review'/de) NOT (('animal experiment'/exp OR 'animal model'/exp OR 'nonhuman'/exp) NOT 'human'/exp) | **497183** |
| #4 | #3 NOT ('conference abstract'/it OR 'editorial'/it OR 'letter'/it OR 'note'/it) NOT (('animal experiment'/exp OR 'animal model'/exp OR 'nonhuman'/exp) NOT 'human'/exp) | **1767** |
| #3 | #1 AND #2 | **2228** |
| #2 | 'cardiovascular risk'/exp OR 'cardiovascular disease'/exp OR 'angiocardiopathy':ti,ab OR 'angiocardiovascular disease':ti,ab OR hypovolemia:ti,ab OR (((cardiovascular OR heart OR cardiac OR coronary) NEAR/3 (complication* OR disorder* OR disturbance* OR lesion* OR syndrome* OR event* OR risk* OR history OR failure OR overload OR dysfunction OR arrest OR aneurysm* OR anomal* OR atheroscleros* OR calcification* OR constriction* OR dissection OR obstruction OR occlusion OR perforation OR thrombos*)):ti,ab) OR 'smoking'/exp OR smoking:ti,ab OR 'obesity'/exp OR 'adipose tissue hyperplasia':ti,ab OR 'adipositas':ti,ab OR 'adiposity':ti,ab OR 'corpulency':ti,ab OR 'fat overload syndrome':ti,ab OR 'obesitas':ti,ab OR 'obesity':ti,ab OR 'overweight':ti,ab OR 'obese patient'/exp OR 'obese':ti,ab OR 'hypercholesterolemia'/exp OR 'cholesteremia':ti,ab OR 'cholesterinemia':ti,ab OR 'cholesterolemia':ti,ab OR 'hypercholesteremia':ti,ab OR 'hypercholesterinaemia':ti,ab OR 'hypercholesterinemia':ti,ab OR 'hypercholesterolaemia':ti,ab OR 'hypercholesterolemia':ti,ab OR 'hypertension'/exp OR 'high blood pressure':ti,ab OR hypertens*:ti,ab OR 'diabetes mellitus'/exp OR 'diabetes':ti,ab OR 'diabetic':ti,ab OR 'insulin resistance'/exp OR 'insulin resistance':ti,ab OR 'nonalcoholic steatohepatitis'/exp OR 'nash (nonalcoholic steatohepatitis)':ti,ab OR 'non alcoholic steato-hepatitis':ti,ab OR 'non-alcoholic steatohepatitis':ti,ab OR 'non-alcoholic steatosis hepatitis':ti,ab OR 'non-alcoholic steatotic hepatitis':ti,ab OR 'nonalcoholic fatty liver inflammation':ti,ab OR 'nonalcoholic steato-hepatitis':ti,ab OR 'nonalcoholic steatohepatitis':ti,ab OR 'nonalcoholic steatosis hepatitis':ti,ab OR 'nonalcoholic steatotic hepatitis':ti,ab OR 'heart arrhythmia'/exp OR 'arrhythmia':ti,ab OR 'ectopic heart rhythm':ti,ab OR 'ectopic rhythm':ti,ab OR 'heart aberrant conduction':ti,ab OR 'arrhytmia':ti,ab OR 'arrythmia':ti,ab OR 'dysrhythmia':ti,ab OR 'heart ectopic beat':ti,ab OR 'heart ectopic ventricle contraction':ti,ab OR 'heart rhythm disorder':ti,ab OR 'atrioventricular junction arrhythmia':ti,ab OR bradycardia:ti,ab OR 'cardiac channelopath*':ti,ab OR 'cardiopulmonary arrest*':ti,ab OR 'commotio cordis':ti,ab OR 'heart fibrillation':ti,ab OR 'heart muscle conduction disturbance':ti,ab OR 'heart palpitation':ti,ab OR 'heart preexcitation':ti,ab OR 'heart proarrhythmia':ti,ab OR 'pacemaker failure*':ti,ab OR parasystole:ti,ab OR tachycardia:ti,ab OR 'acute coronary syndrome':ti,ab OR 'cardiac allograft vasculopathy':ti,ab OR 'coronary bifurcation lesion':ti,ab OR 'coronary subclavian steal syndrome':ti,ab OR 'kounis syndrome':ti,ab OR 'no reflow phenomenon':ti,ab | **5896926** |
| #1 | (('coronavirinae'/exp OR 'coronavirus infection'/de OR coronavirus*:ti,ab,kw OR 'corona virus*':ti,ab,kw OR 'pneumonia virus*':ti,ab,kw OR cov:ti,ab,kw OR ncov:ti,ab,kw) AND (outbreak:ti,ab,kw OR wuhan:ti,ab,kw) OR covid19:ti,ab,kw OR 'covid 19':ti,ab,kw OR ((coronavirus*:ti,ab,kw OR 'corona virus*':ti,ab,kw) AND 2019:ti,ab,kw) OR 'sars cov 2':ti,ab,kw OR sars2:ti,ab,kw OR 'coronavirus*':ti,ab,kw OR 'corona virus*':ti,ab,kw OR 'ncov 2019':ti,ab,kw OR ncov:ti,ab,kw OR 'sars coronavirus 2':ti,ab,kw OR 'sars corona virus 2':ti,ab,kw OR 'severe acute respiratory syndrome cov 2':ti,ab,kw OR 'severe acute respiratory syndrome cov2':ti,ab,kw) AND [2019-2020]/py | **18864** |
