# Supplementary material for: Impact of cardiovascular disease and cardiovascular risk factors in hospitalised COVID-19 patients
Source: Neth Heart J. 2021 Apr 16;29(Suppl 1):13–9. doi: 10.1007/s12471-021-01572-9 (PMC8050809; doi:10.1007/s12471-021-01572-9)
Supplement: Supplementary file 2 — Table S2 Excluded studies [file 12471_2021_1572_MOESM2_ESM.docx]

**Table S2 Excluded studies**

| **Author and year** | **Reason for exclusion** |
| --- | --- |
| Aggarwal 2020 | Wrong comparison: mortality in patients with severe COVID-19 disease and pre-existing history of CVD. Only 3 studies |
| Alqahtani 2020 | Wrong comparison: prevalence of chronic diseases (COPD), no multilevel analysis |
| Cecconi 2020 | Wrong outcome: composite outcome (ICU transfer or death) |
| Galloway 2020 | Wrong outcome: composite outcome (transfer to a critical care unit bed or death) |
| Guan 2020 | Wrong outcome: Only composite endpoint used in multivariate analysis. Same cohort as Chen (2020). |
| Hamer 2020 | Wrong comparison: lifestyle risk factors |
| Hu 2020 | Wrong outcome: composite outcome |
| Huang 2020 | Wrong study design: associations between diabetes and composite outcome composite poor outcome, including mortality, severe COVID-19, ARDS, need for ICU care, and disease progression, no predictive value (no adjustments performed) |
| Imam 2020 | Wrong comparison: imaging findings, medication, laboratory values |
| Jain 2020 | Wrong study design: sample size <200 included |
| Kumar 2020 | Wrong outcome: composite outcome (severe clinical course) |
| Li 2020a | Wrong study design: associations between CVD, hypertension and myocardial injury and mortality, no predictive value (no adjustments performed) |
| Li 2020b | Wrong outcome: severe covid-19 |
| Lippi 2020 | Wrong study design: associations between hypertension and disease severity and mortality, no predictive value (no adjustments performed) |
| Liu 2020 | Wrong outcome: composite outcome (disease severity) |
| Luo 2020 | Article in Chinese |
| Nikpouraghdam 2020 | Wrong comparison: comorbidity as predicting factor (no separate diseases) |
| Parohan 2020 | Wrong study design: associations between comorbidities and mortality, no predictive value (no adjustments performed) |
| Pranata 2020a | Wrong study design: associations between CVD and mortality/poor composite outcome, no predictive value (no adjustments performed) |
| Pranata 2020b | Wrong study design: associations between hypertension and mortality, no predictive value (no adjustments performed) |
| Roncon 2020 | Not searched in Medline. Searched on Diabetes Mellitus |
| Santoso 2020 | Wrong outcome: prognostic effect troponin on outcomes |
| Shi 2020 | Small sample (N<200) |
| Shi 2020a | Wrong comparison: cardiac biomarkers (troponin, CK-MB, MYO) |
| Shi 2020b | Wrong comparison: cardiac injury biomarkers |
| Tamara 2020 | Wrong outcome: severe covid-19 |
| Tian 2020 | Wrong study design: no multilevel analysis |
| Wang 2020a | Wrong outcome: COVID-19 |
| Wang 2020b | Wrong comparison: predictive laboratory model compared to clinical model (age, hypertension, CHD) |
| Zhang 2020a | Wrong outcome: recovery of patients during follow-up |
| Zhang 2020b | Wrong comparison: mortality in hypertensive patients, no prognostic factor (no adjustments performed) |
| Zhang 2020c | Wrong outcome: COVID-19 severity |
| Zhao 2020 | Wrong outcome: severe covid-19 |
| Zheng 2020 | Wrong study design: associations between hypertension, CVD, diabetes and non-critical/critical or mortality, no predictive value (no adjustments performed) |
| Zuin 2020 | Commentary article |
