# Supplementary material for: Impact of cardiovascular disease and cardiovascular risk factors in hospitalised COVID-19 patients
Source: Neth Heart J. 2021 Apr 16;29(Suppl 1):13–9. doi: 10.1007/s12471-021-01572-9 (PMC8050809; doi:10.1007/s12471-021-01572-9)
Supplement: Supplementary file 3 — Table S3 Quality assessment and risk of bias [file 12471_2021_1572_MOESM3_ESM.docx]

**Table S3 Quality assessment and risk of bias– Prognostic factor (PF) studies**

*Based on: QUIPS^A^ (Haydn, 2006; Haydn 2013)*

**Research question: Which independent prognostic factors (cardiovascular risk factors, hypertension, diabetes, cardiovascular disease) strongly predict a poor outcome of COVID-19 infection, independent of other factors?**

| **Study reference**  (first author, year of publication) | **Study participation^1^**  Study sample represents the population of interest on key characteristics?  (high/moderate/low risk of selection bias) | **Study Attrition^2^**  Loss to follow-up not associated with key characteristics (i.e., the study data adequately represent the sample)?  (high/moderate/low risk of attrition bias) | **Prognostic factor measurement^3^**  Was the PF of interest defined and adequately measured?  (high/moderate/low risk of measurement bias related to PF) | **Outcome measurement^3^**  Was the outcome of interest defined and adequately measured?  (high/moderate/low risk of measurement bias related to outcome) | **Study confounding^4^**  Important potential confounders are appropriately accounted for?  (high/moderate/low risk of bias due to confounding) | **Statistical Analysis and Reporting^5^**  Statistical analysis appropriate for the design of the study?  (high/moderate/low risk of bias due to statistical analysis) | **Overall judgment**  *High risk of bias: at least one domain judged to be at high risk of bias.*  *Model development only: high risk of bias.*  Risk of bias: low/moderate/high/unclear |
| --- | --- | --- | --- | --- | --- | --- | --- |
| Chen, 2020 | Unclear (inclusion criteria not clearly described) | Moderate (21% excluded because of incomplete medical records: differences not described) | Unclear (assessment not well described) | High (endpoint for mortality not described: patients could be still under treatment at endpoint of the study) | Low (accounted for age) | Moderate (reason for selection of factors is unclear) | Moderate |
| Cummings, 2020 | High (only COVID-19 patients included who were critically ill with acute hypoxaemic respiratory failure) | Unclear (loss to follow-up not described) | Low | High (some patients were still under treatment at endpoint of the study) | Low (accounted for age) | Moderate (independent variables included in multivariable Cox model considered relevant to in-hospital mortality by the authors. | High |
| Gao, 2020 | Low | Unclear (loss to follow-up not described) | Low | High (patients could be still under treatment at endpoint of the study: not described) | Low (accounted for age) | Unclear (reason of selection of included prognostic factors in multivariable model not described) | Moderate |
| Giacomelli, 2020 | Low | Unclear (loss to follow-up not described) | Low | High (some patients, 10%, were still under treatment at endpoint of the study) | Low (accounted for age) | Low (significant factors univariate analysis included in multivariable analysis) | Moderate |
| Klang, 2020 | Low | High (patients who were still hospitalized during the study period and/or with missing BMI were excluded) | Low | High (22% were still hospitalized at endpoint of the study and were excluded) | Low (accounted for age) | Moderate (all factors included in the multivariate model) | High |
| Palaiodimos, 2020 | Low | Unclear (loss to follow-up not described) | Low | Low | Low (accounted for age) | Low (3 models developed, BMI and age, all the variables with significant univariate associations and addition of clinically significant variables) | Low |
| Petrilli, 2020 | Low | High (patients who were still hospitalized during the study period were censored) | Low | High (mortality after discharge was not measured unless patients was readmitted to the system) | Low (accounted for age) | Low (included all selected predictors based on a priori clinical significance after testing for collinearity using the variance inflation factor) | High |
| Wang, 2020 | Low | Unclear (loss to follow-up not described) | Low | High (54% was still hospitalized at endpoint of the study) | Low (accounted for age) | Low (significant factors univariate analysis included in multivariable analysis) | High |
